# Construction of an Artificial MicroRNA Expression Vector for Simultaneous Inhibition of Multiple Genes in Mammalian Cells

**DOI:** 10.3390/ijms10052158

**Published:** 2009-05-14

**Authors:** Tao Hu, Qiong Fu, Ping Chen, Li Ma, Onsam Sin, Deyin Guo

**Affiliations:** 1 State Key Laboratory of Virology and The Modern Virology Research Centre, College of Life Sciences, Wuhan University, Wuhan 430072, China; E-Mails: hnhutao@sohu.com (T.H.); qiongfu@mail.utexas.edu (Q.F.); mary19840209@126.com (L.M.); soskorea@163.com (O.S.); 2 Department of Pathophysiology, Basic Medical College, Zhengzhou University, Zhengzhou 450001, China; E-Mail: chp7779@sohu.com (P.C.)

**Keywords:** artificial microRNA, expression vector, multiple genes, RNA interference

## Abstract

Recently, artificial microRNA (amiRNA) has become a promising RNA interference (RNAi) technology. Here, we describe a flexible and reliable method for constructing both single- and multi-amiRNA expression vectors. Two universal primers, together with two specific primers carrying the encoding sequence of amiRNA were designed and utilized to synthesize the functional amiRNA cassette through a one-step PCR. With appropriate restriction sites, the synthesized amiRNA cassettes can be cloned into any site of different destination vectors. Using the method, we constructed both single- and multi-amiRNA expression vectors to target three reporter genes, which code firefly luciferase (Fluc), enhanced green fluorescent protein (EGFP) and β-galactosidase (LacZ), respectively. The expressions of three genes were all specifically inhibited by either the corresponding single- or the multi-amiRNA expression vector in 293T cells. And the RNAi efficiency of each amiRNA produced by both single- and multi-amiRNA expression vectors was comparable.

## 1. Introduction

MicroRNAs (miRNAs) are endogenously encoded ~ 22 nt RNAs that can cause target mRNA degradation or translation repression, and play important roles in differentiation, development, cancer, or viral infections [1]. They are most often transcribed by RNA polymerase II as long primary RNA transcripts (pri-miRNAs), which are normally capped at the 5′ end and polyadenylated at the 3′ end [2,3]. Approximately 50% of known miRNA genes are clustered [4], implying that some miRNAs are transcribed as a polycistronic pri-miRNA and processed from a single transcript [5]. The pri-miRNA is processed in the nucleus by Drosha-DGCR8 complex to liberate a 60–70 nt stem loop structure known as precursor miRNA (pre-miRNA) [6,7]. The pre-miRNA is then exported to the cytoplasm by an Exportin 5-dependent mechanism and further processed into a transient ~ 22 bp miRNA:miRNA* duplex (* indicates the passenger strand) by a RNase III-type enzyme Dicer [8–11]. The miRNA:miRNA* duplex is then loaded into the RNA-induced silencing complex (RISC) where the mature miRNA binds to complementary site of target mRNA to inhibit translation [12]. Recently, a significant advance in RNAi technology is the use of artificial microRNAs (amiRNAs).

The amiRNA technology employs the backbone of natural miRNAs to generate designed miRNA that can efficiently and specifically silence the genes of interest. Only the natural miRNA:miRNA* duplex sequence is replaced by the artificial one. Such a design allows amiRNAs to be processed in a similar biogenesis pathway of natural miRNA and results in functional mature amiRNAs. Zeng and colleagues first successfully utilized miR-30 backbone to produce functional amiRNAs and suppress the expression of endogenous human genes [13]. Compared with conventional siRNA and shRNA approaches, the amiRNA technology has the following advantages: 1) Easier to express multiple amiRNAs [14–16]; 2) Easier to achieve tissue-specific RNAi; 3) They confer the ability to control the timing and level of gene silencing; and 4) They provide a correlated marker to track amiRNA-expressing cells [17,18].

However, the difficulty of generating amiRNA expression constructs may limit their wide application. Current methods for constructing amiRNA expression constructs require a special vector carrying the flanking sequence of natural miRNA or the synthesis of long oligonucleotides. The former, such as the pCMV-miR-30 [19], pENTR/CMV-EGFP-miR-30 [20] or SIBR [21] vectors, lack flexibility for employing various miRNA backbones, using different promoters and generating multi-amiRNA expression constructs. The latter, which requires the synthesis of an approximately 96 nt oligonucleotide template with a stem-loop structure [14,22,23], is costly and often suffers from DNA synthesis errors.

In the study, we present a rapid, flexible and reliable method for generating both single- and multi-amiRNA expression vectors. This method only utilizes four short primers (< 60 nt) to synthesize the functional amiRNA cassette through a one-step PCR. The synthesized amiRNA cassette, which contain 5′ cloning sites, 5′ flanking region, amiRNA coding sequence, terminal loop region, amiRNA* coding sequence, 3′ flanking region and 3′ cloning sites, can be cloned individually or in tandem into any destination vector with appropriate sites. With this method, we readily constructed both single- and multi-amiRNA expression vectors to inhibit the expression of EGFP, Fluc and LacZ reporter genes. Our results showed that the expression of all three reporter genes can be specifically suppressed by either corresponding single- or multi-amiRNA expression vector, and the RNAi efficiency of the multi-amiRNA expression vector is comparable to that of the single one.

## 2. Results and Discussion

### 2.1. Construction of amiRNA expression vectors

**Figure 1.**
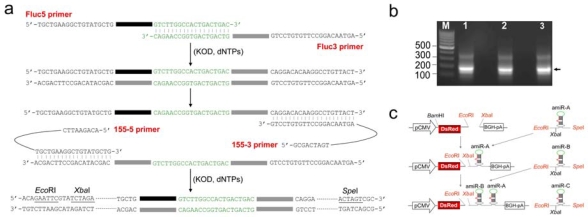
The method for constructing amiRNA expression vector. (a) Schematic representation of the one-step PCR used to synthesize an amiRNA cassette. The black rectangle represents amiRNA. (b) Results of the one-step PCR. The 180 nt DNA fragements are indicated by arrowhead. Lane M, 100 bp DNA ladder; lane 1, amiR-Fluc; lane 2, amiR-EGFP; lane 3, amiR-LacZ. (c) Strategy for generating the multi-amiRNA expression vector.

To validate the method, three reporter genes encoding for Fluc, EGFP and LacZ were selected as targets, and the backbone of miR-155 was employed for the expression of amiRNA. The procedure for synthesizing amiRNA cassette by a one-step PCR is depicted schematically in Figure 1a. In the first several PCR cycles, the two specific primers Fluc5 and Fluc3 were annealed through a 19 bp complimentary region of their 3’ ends, and extended to yield a DNA fragment about 100 bp by KOD DNA polymerase. The overlap-extended DNA fragment was then amplified by two universal primers 155-5 and 155-3 to produce the amiRNA cassette against Fluc (amiR-Fluc) as designed. The 19-nt terminal loop region was used for annealing of two specific primers, and provided a proper annealing temperature for specific amplification. Previous research showed that during construction of miR-30-based shRNA libraries [22], 97-nt DNA templates for each silencing trigger were designed to be amplified by universal primers, but only 25 – 60% of the clones had correct shRNA sequences. Another research also indicated that up to 50% of these ‘positive’ recombinant clones contained substitutions and deletions when using Taq polymerase to construct shRNA vector [24]. However, in the method provided here, all four primers for synthesis of amiRNA cassette are shorter than 60 nt in length. The cost and possibility of errors arising from chemical synthesis of long oligonucleotides are greatly reduced. At the same time, the proofreading KOD DNA polymerase was also employed to reduce the mutation rates of PCR amplification.

The resulting PCR products were analyzed on 1.2% agarose gel. Figure 1b shows that the synthesized amiRNA cassettes have an expected molecular size of about 180 bp. These PCR products were then recovered by the glass beads method, which provides high extraction efficiency for small DNA fragments. Subsequently, amiRNA cassettes against Fluc, EGFP (amiR-EGFP) or LacZ (amiR-LacZ) were inserted individually or in tandem into the pDsRed vector via unique but compatible restriction sites (Figure 1c). The resulting vectors were named pDsRed-amiR-Fluc, pDsRed-amiR-EGFP, pDsRed-amiR-LacZ, and pDsRed-amiR-ZGF (contains the cassettes of amiR-LacZ, amiR-EGFP and amiR-Fluc in tandem), respectively. In these vectors, the red fluorescence gene (DsRed) tightly linked to the amiRNA cassette allows us to track the efficiency of transfection. All amiRNA expression vectors were verified by sequencing with BGH reverse primer. Using this method, we obtained at least two correct constructs out of three for each amiRNA expression vector.

### 2.2. Suppression of firefly luciferase expression by amiR-Fluc expression vectors

To test whether these amiRNA expression vectors would be functionally effective, RNAi efficiencies against corresponding target genes were evaluated by transient transfection assays. Firefly luciferase expression vector pGL3-CMV was co-transfected with amiR-Fluc expression vector pDsRed-amiR-Fluc or pDsRed-amiR-ZGF into 293T cells.

**Figure 2.**
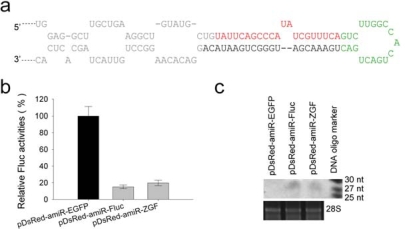
Suppression of firefly luciferase expression by either single amiRNA expression vector pDsRed-amiR-Fluc or multi-amiRNA expression vector pDsRed-amiR-ZGF in 293T cells. (a) The structure and sequence of pri-amiR-Fluc. Green letters represent the terminal loop region; red letters represent the amiR-Fluc sequence; gray letters represent the flanking sequence. (b) Luciferase expression vector pGL-CMV (50 ng) was co-transfected into 293T cells with 150 ng amiR-Fluc expression vectors or pDsRed-amiR-EGFP. Firefly luciferase activities were measured 24 hours after transfection. The luciferase activity in the presence of pDsRed-amiR-EGFP was set at 100%. Error bars represent the standard deviation from three independent experiments (*p* < 0.001 for both amiR-Fluc expression vectors when compared to the control vector). (c) Detection of amiR-Fluc expression by Northern blotting. The amiRNAs migrated slightly slower than DNA markers and at an approximate size of 28 nt DNA oligonucleotide. The 28S rRNA served as an internal control for equal RNA loading.

The structure and sequence of pri-amiR-Fluc is shown in Figure 2a. About 24 h after transfection, the luciferase activity was measured. The activity in control cells co-transfected with pGL3-CMV and pDsRed-miR-EGFP was set as 100%. Figure 2b shows that pDsRed-amiR-Fluc and pDsRed-amiR-ZGF all significantly reduced luciferase activity. The Northern blot analyses using a probe complementary to amiR-Fluc sequence also confirmed that both pDsRed-amiR-Fluc and pDsRed-amiR-ZGF produced the mature amiR-Fluc of about 22 nt in transfected cells (Figure 2c).

### 2.3. Efficient suppression of EGFP expression by amiR-EGFP expression vectors

We next examined the inhibition effect on EGFP expression induced by amiR-EGFP expression vectors. The pDsRed-amiR-EGFP or pDsRed-amiR-ZGF was co-transfected with pEGFP-C1 vector into 293T cells, and the RNAi effect was evaluated by fluorescent microscopy and Western blotting.

**Figure 3.**
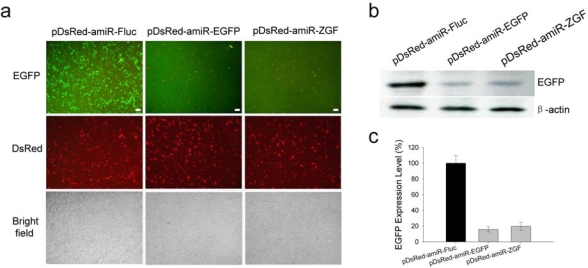
Efficient suppression of EGFP expression by pDsRed-amiR-EGFP or pDsRed-amiR-ZGF in 293T cells. (a) EGFP expression vector pEGFP-C1 (100 ng) was co-transfected with 100 ng amiR-EGFP expression vectors or pDsRed-amiR-Fluc into 293T cells. EGFP expression was detected by fluorescence microscopy. (b) EGFP expression was analyzed by Western blotting. The Western blotting shows a representative result from three independent experiments. (c) After normalizing to β-actin, the inhibition level induced by pDsRed-amiR-EGFP or pDsRed-amiR-ZGF was assessed by densitometry. Normalized values (mean ± SD) from three independent Western blotting experiments are shown (*p* < 0.001 for both amiR-EGFP expression vectors when compared to the control vector).

As shown in Figure 3a, the expression of EGFP was obviously suppressed by either pDsRed-amiR-EGFP or pDsRed-amiR-ZGF. The observation under fluorescent microscope was further confirmed by the Western blotting result (Figure 3b). After normalizing to β-actin, EGFP expression level in the pDsRed-amiR-EGFP- or pDsRed-amiR-ZGF-transfected 293T cells was only 16% or 19% of the control (pDsRed-amiR-Fluc-transfected) cells, as determined by densitometry (Figure 3c). At the same time, the results of red fluorescence observations suggested that the marker protein DsRed were expressed at reasonable levels in all cells transfected with amiRNA expression vectors (Figure 3a). Such arrangement was also proved to be effective by other reports. However, when the ORF of DsRed was placed at the 3′ end of amiRNA cassette, the DsRed was poorly expressed (data not shown). Thus, placing the reporter gene at 5′ end of amiRNA cassette would be a better approach to co-express amiRNA along with the marker protein. In addition, a strategy to express amiRNA from an intron was also used to improve the marker gene expression [17,18].

### 2.4. Efficient inhibition of β-galactosidase expression by amiR-LacZ expression vectors

**Figure 4.**
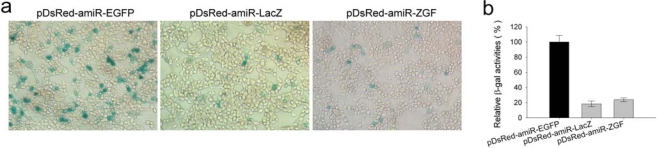
Inhibition of β-galactosidase expression by pDsRed-amiR-LacZ or pDsRed-amiR-ZGF in 293T cells. (a) The pSV-β-Galactosidase expression vector (100 ng) was co-transfected with 100 ng amiR-LacZ expression vectors or pDsRed-amiR-EGFP into 293T cells. The β-galactosidase expression was detected by X-gal staining. (b) β-Galactosidase activity was further determined by the Beta-Glo assay system kit (Promega) according to the manufacturer’s instructions. A background value was subtracted from each reading before comparison. The β-galactosidase activity in the presence of pDsRed-amiR-EGFP was set at 100%. Error bars represent standard deviation from three independent experiments (*p* < 0.001 for both amiR-LacZ expression vectors when compared to the control vector).

The expression of LacZ reporter gene was also specifically suppressed by either pDsRed-amiR-LacZ or pDsRed-amiR-ZGF in 293T cells (Figure 4a), and their RNAi efficiencies were 79% and 74%, respectively (Figure 4b). Taken together, all amiRNA expression vectors constructed in the study were functionally effective, and the expressions of all three reporter genes were specifically suppressed by either corresponding single- or multi-amiRNA expression vector. These results shows that multiple amiRNAs can be co-expressed from a single transcript, and RNAi efficiency of each is comparable to that produced by the single amiRNA expression vector.

Endogenous pri-miRNAs share some similar structural characteristics that comprise a stem, a terminal loop, long flanking sequences, and some internal loops or bulges, which likely contribute to an efficient processing in miRNA pathway [25,26]. The sequence flanking pre-miRNA has been proven critical for pri-miRNA to be processed into pre-miRNA by Drosha-DGCR8 complex [27,28]. As reported for miR-223, 40 nt of flanking sequence on each side of pre-miR-223 are necessary for the maturation of miR-223 [29]. Chung *et al*. delimited a smallest fully functional fragment of 108 nt (BIC 134–241) that is sufficient to produce mature miR-155 in mammalian cells [21]. The miR-30 can be generated correctly and efficiently when the pre-miRNA is flanked by 22 nt flanking sequences at its 5’ side and 15 nt at its 3’ side [18]. Sun *et al*. also used an extended miR-30 hairpin of 118 nt as a backbone for amiRNA expression cassette [14]. Based on these previous results, we reasoned that 40 nt flanking sequence on each side of pre-miRNA are essential and sufficient for the processing and maturation of most miRNAs. Therefore, a functional amiRNA cassette should usually contain 5′ flanking region of at least 40 nt from natural pri-miRNA, encoding sequence of amiRNA, the terminal loop region, coding sequence of amiRNA* and 3′ flanking region of at least 40 nt from natural pri-miRNA, in the order of a 5′ to 3′ direction. Appropriate restriction sites can be introduced at both ends of cassette. This might be considered as a general rule for designing the amiRNA or even natural miRNA cassette. According to this rule, a functional amiRNA cassette plus cloning sites would be approximately 170 bp in length. With appropriate sites, the synthesized amiRNA cassette can be inserted into any site of different expression vectors, which would greatly enhance the flexibility to construct amiRNA expression vectors.

Many endogenous miRNA genes are found in close proximity to others. These natural miRNAs can be generated from a single polycistronic primary transcript [30], and several groups have demonstrated that some artificial miRNAs could be expressed from one single RNA transcript. It’s has been observed that multiple copies of an amiRNA expressed from a single polycistronic transcript could enhance the RNAi effect [16,21]. But surprisingly, it has also been reported that miRNA yields and gene silencing effects were substantially increased when two distinct amiRNAs co-expressed in tandem from a single transcript [14]. In our study, the expression of all three reporter genes can be suppressed at a high level by either corresponding single- or multi-amiRNA expression vector, and the silencing effects are comparable between the single and multiple one. Therefore, by using amiRNA technology, RNA silencing of multiple genes can be readily and simultaneously achieved.

## 3. Experimental Section

### 3.1. Synthesis of amiRNA cassette by one-step PCR

Three reporter genes coding for Fluc, EGFP and LacZ were selected to test the amiRNA-mediated RNAi efficiency. The amiR-Fluc was designed according to a previous report [17]. The amiR-EGFP and amiR-LacZ were designed using the Block-it RNAi designer (http://rnaidesigner.invitrogen.com). The amiRNAs target sequences are as follows: coding regions 200 – 220 (5′-TGAAACGATATGGGCTGAATA-3′) for Fluc; 123 – 143 (5′-CAAGCTGACCCTGAAGTTCAT-3′) for EGFP; 642 – 662 (5′-GACTACACAAATCAGCGATTT-3′) for LacZ. Two universal primers and three pairs of specific primers were designed and then synthesized by Invitrogen. The universal primer 155-5 includes sequence of 5′-flanking region of pre-miR-155, *Eco*RI and *Xba*I restriction sites. The universal primer 155-3 includes reverse complement sequence of 3′-flanking region of pre-miR-155 and SpeI site. The specific primer pairs Fluc1/Fluc2, EGFP1/EGFP2 and LacZ1/LacZ2 carry the coding sequence of amiRNA against the corresponding target gene and have a 19 nt complementary overlap at their 3′ ends. Sequences of all primers are listed in Table 1. The two universal primers and one pair of specific primers were utilized to synthesize an amiRNA cassette by one-step PCR. Schematic representation of one-step PCR is shown in Figure 1a.

PCR amplification was performed in a 50 μL volume containing 1 x PCR reaction buffer, 25 pmol each of universal primers, 5 pmol of each specific primer, 1 mM MgSO_4_, 0.2 mM dNTPs and 1 unit KOD-Plus DNA polymerase (TOYOBO). The PCR program was as follows: 94 °C for 3 min, 35 cycles of 15 sec at 94 °C, 30 sec at 55 °C, 15 sec at 68 °C and a final extension for 8 min at 68 °C. The PCR products were separated on a 1.2% agarose gel stained with ethidium bromide and compared to 100 bp DNA ladder (Fermentas).

### 3.2. Plasmid construction

The coding sequence of red fluorescent protein (DsRed) was cleaved from pDsRed2 vector (Clontech) with *Bam*HI and *Eco*RI, and then inserted into pVAX1 vector (Invitrogen) to generate vector pDsRed. The synthesized amiR-Fluc, amiR-EGFP or amiR-LacZ cassette was digested with *Eco*RI and *Spe*I, and then cloned into the pDsRed vector, which was digested with *Eco*RI and *Xba*I (compatible end with *Spe*I), to generate pDsRed-amiRFluc, pDsRed-amiREGFP and pDsRed-amiRLacZ vectors. The strategy for generating multi-amiRNA expression vector is illustrated in Figure 1c. These constructs can be identified by sequencing with BGH reverse primer.

### 3.3. Cell culture and DNA transfection

HEK 293T cells were plated in 96-well plates at 1.5 × 10^4^ cells per well and grown as a monolayer in DMEM (Invitrogen) supplemented with 10% fetal calf serum at 37 °C and 5% CO_2_. Cells at 70% confluence were co-transfected with the indicated amounts of plasmids using Lipofectamine 2,000 reagent (Invitrogen) according to the manufacturer's instructions.

### 3.4. Reporter assays

Firefly luciferase activity assay was performed 24 hours after transfection using a Steady-Glo Luciferase Assay System Kit (Promega), and chemiluminescence was measured in a luminometer (Tecan). The ß-galactosidase activity was quantified by using the Beta-Glo Assay System (Promega) in accordance with the manufacturer’s protocol. The X-gal staining of cells was performed as described previously [31].

### 3.5. Western blotting

Western blotting was performed as described previously [32]. EGFP expression was detected by using mouse monoclonal anti-GFP antibody (Lab Vision) as primary antibodies, and probed with a goat anti–mouse IgG horseradish peroxidase conjugate (Pierce).

### 3.6. Northern blotting

Twenty-four hours after transfection, total RNA was extracted using TRIzol reagent (Invitrogen) following the manufacturer’s protocol. Thirty micrograms of total RNA was separated by electrophoresis on a 15% denaturing polyacrylamide gel, and blotted onto Hybond N^+^ membranes. The 25-nt, 27-nt and 30-nt DNA oligonucletides were also loaded on the same gel to provide molecular weight markers. RNA was immobilized by UV crosslinking. Hybridization was carried out at 37 °C using Hybridization Buffer (Innogent). The amiR-Fluc was probed with a digoxigenin-labeled 21-nt DNA (5′-TGAAACGATATGGGCTGAATA-3′) using DIG nucleic acid detection kit II (Innogent).

## 4. Conclusions

In summary, we propose a general rule for amiRNA design. According to this rule, a one-step PCR-based method was developed to construct both single- and multi-amiRNA expression vectors. All amiRNA expression vectors constructed in this study could specifically inhibit the expression of corresponding target genes in mammalian cells. The RNAi efficiency of each amiRNA produced by the multi-amiRNA vector is comparable to that of the single one. With the method, we constructed more than 100 amiR-Luc expression vectors using different expression patterns or with different pri-miRNA structures to further optimize this amiRNA expression system. It should also be mentioned that this method can be extended to employ other miRNA backbones. In our lab, the backbones of miR-16, miR-206, and miR-331 were all successfully employed to express functional amiRNAs (unpublished data). These results show that our method for constructing amiRNA expression vector is highly flexible and reliable and will facilitate the application of amiRNA technology in basic research and gene therapy. In addition, the multi-amiRNA expression vector also make it more convenient and efficient to increase the silencing efficiency of a target gene or a virus, to knockdown the expression of multiple related genes, or to inhibit the replication of two or more viruses simultaneously.

## Figures and Tables

**Table 1.** Oligonucleotides used in this paper.

**Name**	**Sequence (5’-3’)**
155-5	ACAGAATTCGACTCTAGAATCCTCTGGCTGCTGGAGGCTTGCTGAAGGCTGTATGCTG
155-3	GCGACTAGTACGGTGGCCATTTGTTCCATGTGAGTGCTAGTAACAGGCCTTGTGTCCTG
Fluc5	TGCTGAAGGCTGTATGCTGTATTCAGCCCATATCGTTTCAGTCTTGGCCACTGACTGAC
Fluc3	AGTAACAGGCCTTGTGTCCTGTATTCAGCCCATCGTTTCAGTCAGTCAGTGGCCAAGAC
EGFP5	TGCTGAAGGCTGTATGCTGATGAACTTCAGGGTCAGCTTGGTCTTGGCCACTGACTGAC
EGFP3	AGTAACAGGCCTTGTGTCCTGATGAACTTCAGTCAGCTTGGTCAGTCAGTGGCCAAGAC
LacZ5	TGCTGAAGGCTGTATGCTGAAATCGCTGATTTGTGTAGTCGTCTTGGCCACTGACTGAC
LacZ3	AGTAACAGGCCTTGTGTCCTGAAATCGCTGATGTGTAGTCGTCAGTCAGTGGCCAAGAC

* The underlined letters present the sequences of amiRNAs.
